# Lipoprotein-Associated Oxidative Stress: A New Twist to the Postprandial Hypothesis

**DOI:** 10.3390/ijms16010401

**Published:** 2014-12-26

**Authors:** Ngoc-Anh Le

**Affiliations:** Fellow, American Heart Association, Laboratory Director, Biomarker Core Laboratory, Atlanta Research and Education Foundation, Atlanta VA Medical Center, Decatur, GA 30033, USA; E-Mail: Anh.Le@va.gov; Tel.: +1-404-307-3222

**Keywords:** oxidative stress, reactive oxygen species, lipoproteins, postprandial lipemia

## Abstract

Oxidative stress is recognized as one of the primary processes underlying the initiation and progression of atherosclerotic vascular disease. Under physiological conditions, the balance between reactive oxygen species (ROS) generation and ROS scavenging is tightly controlled. As part of normal cellular metabolism, regulated oxidative stress is responsible for a variety of cellular responses. Excess generation of ROS that could not be compensated by antioxidant system has been suggested to be responsible for a number of pathological conditions. Due to their short biological half-lives, direct measurement of ROS is not available and surrogate measures are commonly used. Plasma lipoproteins, by virtue of their close interactions with endothelial cells in the vasculature and the susceptibility of their surface lipids to oxidative modification, are perfect biological sensors of oxidative stress in the arterial wall. In particular, with each consumed meal, triglyceride-rich lipoproteins, secreted by the intestine into the circulation, are responsible for the delivery of 20–40 grams of fat to the peripheral tissues. This flux of dietary lipids is accompanied by concomitant increases in glucose, insulin and other meal-associated metabolites. The contribution of postprandial lipemia to the pathogenesis of atherosclerosis has been previously suggested by several lines of investigation. We have extended this hypothesis by demonstrating the acute generation of oxidative epitopes on plasma lipoproteins as well as transient changes in the oxidative susceptibility of plasma lipoproteins.

## 1. Oxidative Stress and Reactive Oxygen Species (ROS)

Reactive oxygen species (ROS) are produced from molecular oxygen as a result of normal cellular function and can be classified as either free radicals or nonradicals. Some of the common free radicals are superoxide anion (O_2_**^−^**·), hydroxyl radical (·OH), and hydrogen peroxide (H_2_O_2_) as a nonradical ROS. Superoxide anions could be generated *in vivo* by a number of processes involving molecules such as (NAD(P)H) oxidase, xanthine oxidase, or by the electron transport system of mitochondria. Superoxide anions can also be converted to hydrogen peroxide (an example of a nonradical species) by superoxide dismutase (SOD). The hydroxyl radical is the most reactive form of ROS and can initiate lipid peroxidation by attacking polyunsaturated fatty acids (PUFA). Other oxygen-derived free radicals such as peroxyl radicals (ROO··), in particular hydroxyperoxyl radical (HOO··), could also contribute to fatty acid peroxidation.

Heavy metal ions can also interact with superoxide anions to form hydrogen peroxide (Haber–Weiss reaction), which can subsequently be converted to OH radicals (Fenton reaction). In the presence of polyunsaturated fatty acids, a lipid radical could be formed (seeding process). The lipid radical can subsequently react with oxygen to produce peroxyl radicals, which can initiate a chain reaction resulting in the formation of lipid hydroperoxides. If these radicals are not quenched by antioxidants, this process will propagate until all of the PUFA molecules are modified. Lipid hydroperoxides, being very unstable, are rapidly converted to secondary products such as malondialdehydes (MDA) and 4-hydroxy-2,3-nonenals (HNE).

While most of the enzymes responsible for the generation of ROS are intracellular [1], excess ROS can find its way into the extracellular space and affect plasma metabolites. Most of these oxidizing metabolites have extremely short biological half-lives and are difficult to detect *in vivo*. Plasma levels of secondary end-products, such as isoprostanes, carbonyls, MDA and HNE-modified compounds have been used as surrogate indicators of oxidative stress. In the following sections we will present evidence suggesting that plasma lipoproteins may not only serve as sensitive physiologic biosensors of oxidative stress but also contribute to the propagation of oxidative damage throughout the vasculature [2].

## 2. Oxidized Low-Density Lipoproteins (LDL) and Atherosclerosis

Atherosclerosis is recognized as an inflammatory process characterized by the accumulation of cholesterol in macrophages trapped in the sub-endothelium and extracellular matrix [3,4]. While epidemiologic and intervention studies have implicated plasma low-density lipoproteins (LDL) as the primary source of cholesterol, *in vitro* studies with cell cultures would suggest that LDL in its native form could not contribute to this accumulation, and that only chemically modified LDL could lead to the formation of foam cells [5,6,7]. Oxidatively modified LDL is one form of modified LDL that has been demonstrated to be present *in vivo* [8], specifically in atherosclerotic lesions [9,10], and has been implicated in the pathogenesis of atherosclerotic diseases [11,12,13,14]. These observations and other data from animal and human studies are summarized in the oxidation hypothesis of atherosclerosis [15,16,17,18]. According to this hypothesis, native LDL does not accumulate in monocyte-derived macrophages, independent of concentration. LDL has to be modified before it can be taken up via scavenger receptors and initiate the lipid accumulation process. Delayed clearance of LDL in individuals with hypercholesterolemia increases the propensity for circulating LDL to be modified and thus contribute to the initiation and progression of atherosclerosis [17]. Indeed individuals with risk factors for cardiovascular disease tend to have higher levels of oxidized LDL (oxLDL) [19] and high concentrations of oxLDL predict future cardiovascular events [20]. Under this hypothesis, the presence of a pro-inflammatory state serves as fuel to accelerate the oxidative modification of LDL [4,21,22,23,24].

Alternatives to the oxidation hypothesis of atherosclerosis include the response-to-injury hypothesis which suggests that the initial step in atherogenesis is endothelial denudation resulting in compensatory responses that alter normal vascular function [25]. The response-to-retention hypothesis is based on the observation that, due its size, LDL particles can be delivered to the subendothelium by transcytosis, retained within the arterial wall, forming microaggregates that are subsequently taken up by macrophages and smooth muscle cells [26,27].

While the impact of oxLDL in atherogenesis is well recognized, the exact process for the *in vivo* modification of plasma LDL remains unclear. Given the endogenous antioxidant defenses present in human blood [28], it has been suggested that plasma LDL must be trapped in some microenvironment with excess oxidants for the modification to occur. According to the oxidation hypothesis, LDL, being smaller in size, is trapped in the subendothelium where it could be modified by resident macrophages. Once modified, oxLDL can play a central role in progression of atherosclerosis by (i) Promoting the recruitment of circulating monocytes into the intimal space via monocyte chemotactic protein-1, MCP-1 [29]; (ii) Inhibiting the movement of resident macrophages to leave the intima [30]; (iii) Accelerating the rate of uptake and accumulation of lipoproteins resulting in the formation of foam cells [5,31,32,33,34]; and (iv) Being cytotoxic to endothelial cells leading to loss of endothelial integrity [32,33,34].

## 3. Biomarkers of Oxidative Modification

Oxidative stress occurs when cellular antioxidant defense processes are inadequate to inactivate reactive oxygen species (ROS) [35] and reactive nitrogen species (RNS) [36], either because of excess production of ROS/RNS or a dysfunctional antioxidant system. Direct detection of ROS/RNS remains a challenge due to the instability of these reactive species and the need of for special equipment to detect ROS/RNS by electron spin resonance [37]. Surrogate markers of oxidation include stable metabolites (e.g., nitrate or nitrite) and oxidation target products such as lipid peroxidation end products [38,39] and oxidized proteins [40,41].

Exposure of PUFA to ROS or RNS may lead to lipid peroxidation. Lipid peroxidation, in turn, can result in changes in membrane properties that might affect fluidity and activity of membrane-bound receptors as well as generation of secondary oxidized products that are chemically reactive. Lipid peroxidation generates a number of relatively stable endproducts such as reactive aldehydes, e.g., malondialdehyde (MDA) 4-hydroxy-2-nonenal (HNE) and 2-propenal (acrolein) [42] and isoprostanes [38,43].

MDA is produced by peroxidative decomposition of polyunsaturated lipids and reacts mainly with lysine residues of plasma proteins. MDA-modified proteins are characteristically immunogenic and autoantibodies against MDA-modified proteins have been detected *in vivo* [44]. Increased levels of MDA have been reported in plasma and atherosclerotic plaques of patients with type 2 diabetes mellitus (T2DM) [45]. Adducts of apoB-100 lysine residues with MDA and HNE have been characterized in human atherosclerotic lesions [46]. Apolipoprotein B-100 (apoB-100), constitutes 95% of the protein component in LDLHNE is generated from free radical modification of ω6 polyunsaturated fatty acids and is a toxic second messenger of oxygen free radicals [42]. It can undergo reactions with proteins, peptides, phospholipids, and nucleic acids resulting in changes in a wide range of biological activities [47]. Acrolein is present in environmental sources, specifically cigarette smoke. Acrolein reacts with lysine residues of apolipoprotein A-I (apoA-I), the primary structural apolipoprotein of plasma high-density lipoproteins (HDL) resulting in impairment of the function of HDL in cholesterol efflux and acrolein-modified apoA-I has been localized in human atherosclerotic plaques [48].

F_2_-Isoprostanes (F_2_-IsoP) are a family of prostaglandin-like compounds generated *in vivo* by free radical-catalyzed non-enzymatic peroxidation of esterified arachidonic acid, released into the circulation by phospholipases and subsequently excreted in the urine [38,43]. One form of F_2_-IsoPs, 8-iso-PGF2a, is found in plasma primarily esterified to lipids with only a minor component as free acids. F_2_-IsoPs are chemically stable *in vivo* but are rapidly metabolized and excreted as free acids in the urine.

Just as with lipid peroxidation, a number of modifications could occur. Under conditions of moderate oxidative stress, oxidation of cysteine residues lead to the formation of mixed disulfides between protein thiol groups and GSH, a process referred to as *S*-glutathionylation. GSSG:GSH ratio (glutathione disulfide versus reduced glutathione) in blood has been used as an indicator of oxidative status in humans [49]. *S*-glutathionylated proteins have been assessed as potential biomarkers of oxidative/nitrosative stress in human diseases. For instance, glutathionylated hemoglobin has been reported to be increased in patients with diabetes, hyperlipidemia, and in patients on hemodialysis or peritoneal dialysis [50].

Tyrosine moieties on proteins can be modified resulting in the formation of 3-nitrotyrosine (NO_2_-Tyr), 3-chlorotyrosine (Cl-Tyr), or 3-bromotyrosine [51,52]. High levels of HDL apoA-I modified with NO_2_-Tyr and Cl-Tyr have been reported [53] as isolated from whole plasma or from atherosclerotic plaques [54,55].

## 4. Oxidative Susceptibility of Plasma Lipoproteins

Once the concept that (1) oxLDL does play a critical role in the initiation and progression of atherosclerosis and (2) LDL must be retained in an oxidant-rich microenvironment for oxidative modification to occur, is accepted, the actual process of lipoprotein oxidation remains to be understood. Several forms of LDL oxidation have been characterized in animal models [56,57] as well as in human subjects [9,10,14,29].

**Figure 1:**
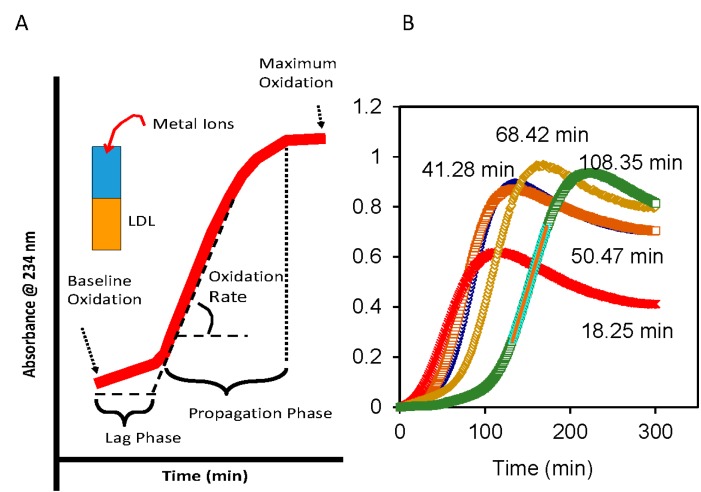
Oxidative susceptibility of plasma Low-Density Lipoproteins (LDL) in the presence of Cu^2+^ as catalyst. (**A**) Kinetics of conjugated dienes formation as assessed by absorbance (234 nm) include three phases: Lag phase, propagation phase and degradation phase. Degradation phase is characterized by loss of signal after the maximum oxidation is reached; and (**B**) Under comparable incubation conditions, the lag phases for LDL isolated from fasting plasma of different individuals can vary widely from 18 to 108 min.

The continuous monitoring of the formation of lipid hydroperoxides on isolated LDL in the presence of Cu^2+^, as detected at 234 nm, (Figure 1A) has proven to be a useful assay for oxidative susceptibility [58]. For this assay LDL is isolated from freshly collected plasma either by density ultracentrifugation (d: 1.020–1.063 g/mL) or by a combination of ultracentrifugation and column chromatography [59]. When controlled for the ratio of LDL-cholesterol and Cu^2+^, plasma LDL isolated from different individuals could have a wide range of lag times (Figure 1B), with longer lag time being indicative of reduced oxidative susceptibility. The lag time for LDL depends on the ratio of Cu^2+^ to LDL-cholesterol (LDL-C) and temperature of the incubation. Shorter lag time would be obtained with either higher concentrations of Cu^2+^ or higher temperature. In our laboratory, lag times were most reproducible at room temperature and 9 mmol of Cu^2+^ with 45 mg of LDL-C [59].

**Figure 2:**
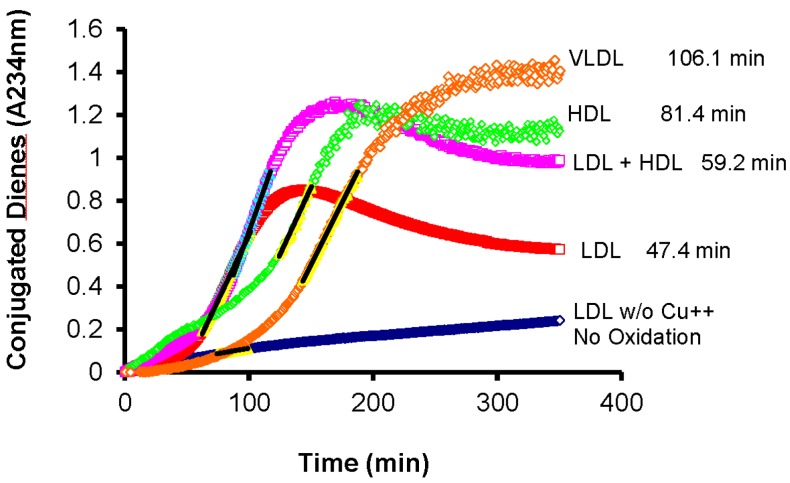
All lipoproteins isolated from fasting plasma are susceptible to oxidative modification in the presence of Cu^2+^ as a catalyst. (VLDL: Very-low density lipoproteins; LDL: Low-density lipoproteins; HDL: High-density lipoproteins; See [59] for details on the isolation procedure and incubation conditions).

It should be noted that this oxidative process is not limited to plasma LDL but all plasma lipoproteins are susceptible to oxidative modification (Figure 2).

Several investigators have reported on both the variability in lag times among individuals as well as the fact that all plasma lipoproteins may be subjected to oxidative modification using similar conditions. For instance, McEneny *et al.* [60], have reported that VLDL isolated from fasting plasma of patients with type 2 diabetes mellitus (T2DM) have significantly shorter lag times, *i.e.*, more susceptible to oxidative modification, than VLDL isolated from healthy control subjects. They also noted that different VLDL subfractions isolated by density ultracentrifugation from the same plasma may also have different lag times, with the smaller particles being more susceptible to oxidative modification [60]. This is in spite of the fact that the subpopulation of larger VLDL tends to have more pre-formed lipid hydroperoxides than the smaller VLDL subpopulation [60]. The simple explanation is that smaller particles have less space for antioxidants, both on the surface and in the core, while larger particles having more lipids would have more lipid hydroperoxides per particle. The degree of oxidizability has also been shown to correlate with the cytotoxicity of the lipoproteins when exposed to endothelial cells [34].

Oxidative susceptibility of plasma LDL as assessed by the lag time of Cu^2+^-induced formation of conjugated dienes (Figure 1A) has been reported to predict the presence of coronary artery disease (CAD), independent of traditional risk factors [61]. Reduced lag times have been reported in patients with cardiovascular disease (CVD) risk, including patients with ischemic stroke [60,62], T2DM [60], and patients with chronic renal failure undergoing regular hemodialysis [63]. Longer lag time, *i.e.*, resistance to oxidation, was associated with increased β-carotene and vitamin E contents in the LDL particle [64]. Oxidative susceptibility of plasma LDL could be affected by daily activities such as exercise [65] and diet [66]. Diets high in polyunsaturated fatty acid, e.g., linolenic acid (18:2) resulted in lower resistance to oxidation as compared to diets with monounsaturated fatty acid, oleic acid (18:1) [67,68,69].

## 5. Plasma Lipoproteins and the Arterial Wall: Fasting *versus* Postprandial

While the focus of cardiovascular risk has been on LDL which accounts for 60% of the cholesterol concentration in plasma, triglycerides transported by the triglyceride-rich lipoproteins (TRL) can contribute significantly to the PUFA flux. For a typical individual with an LDL-C concentration of 130 mg/dL, a plasma volume of 2000 mL, and a fractional clearance rate of 0.4 pool/day, only 1 gram of cholesterol is transported during a 24-h period. For the same individual with a caloric intake of 2000 kcal/day and 30% from fat, the TRL is responsible for the delivery of 55 grams of triglycerides (TG) per 24 h. In addition to this flux of dietary TG carried by the intestinal chylomicrons, there is an additional 8–10 grams of TG/day that is carried by the very-low density lipoproteins (VLDL) secreted by the liver.

In the circulation, TRL becomes attached to the arterial wall via heparan sulfate proteoglycans and remains attached during interactions with lipoprotein lipase (LPL) which is responsible for the hydrolysis of the TG cargo [70,71]. The released free fatty acids (FFA) and monoglycerides can now diffuse through the endothelial cell layer and are re-constituted as TG for storage [72]. Available *in vitro* data suggests that, as the concentration of FFA in the immediate microenvironment reaches a critical level, LPL action is interrupted, and the partially hydrolyzed TRL is released back into the circulation and moves on to the next site [73,74]. An alternate hypothesis is based on the activation/inhibition of LPL by specific apolipoproteins present on TRL. While apolipoprotein B (apoB) is the primary structural apolipoprotein for TRL, the interactions of these TG-rich lipoproteins with LPL are modulated by two other smaller apolipoproteins, apoC-II and apoC-III [75,76]. It has been suggested that TG hydrolysis will proceed with apoC-II as the required activator. However, with the loss of core TG, apoC-III may redistribute on the surface of the particle and interfere with the action of LPL, causing the particle to be released into the circulation, and thus allowing interactions of partially delipidated TRL with other LPL molecules at downstream sites [77,78,79].

It is our hypothesis that during the interactions of TRL with LPL that is anchored on the arterial wall, plasma lipoproteins could be seeded with ROS generated in the subendothelium. This transfer of oxidative epitopes onto plasma lipoproteins would account for the acute and transient reduction in circulating autoantibodies against oxidatively modified LDL observed during postprandial lipemia in patients with diseased endothelium [80]. In normolipidemic controls without excess ROS, there would be negligible transfer of oxidative epitopes onto plasma lipoproteins, and reduction in circulating autoantibodies against oxLDL would not be observed. [80]. This meal-induced response is also specific for PUFA which is highly susceptible to oxidative modification and could not be demonstrated when test meals containing other fatty acids are used [81].

**Figure 3:**
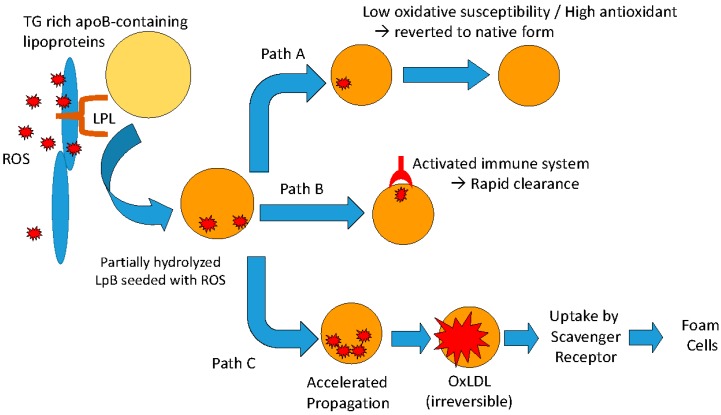
Possible metabolic fates of apoB-containing lipoproteins that have been seeded with reactive oxygen species (ROS) following interactions with lipoprotein lipase anchored along the arterial wall.

A corollary to this hypothesis addresses the oxidative susceptibility of the lipoproteins in the circulation. In the current scheme (Figure 3), once the lipoprotein particle is seeded with ROS it can follow a number of possible metabolic pathways [82].

Path A: In the normal individual, ROS newly transferred onto the plasma lipoprotein particle is rapidly quenched by antioxidants present on the particle and the lipoprotein particle is restored to its native state. One would expect that a lipoprotein particle that is resistant to oxidative modification, *i.e.*, longer lag time (low oxidative susceptibility), would be more likely to be restored to its native state.

Path B: The lipoprotein particle seeded with ROS may be recognized by autoantibodies specific of oxidized epitopes and is rapidly cleared from the circulation. This would be analogous to the case of Watanabe Heritable HyperLipidemia (WHHL) which has been reported to have reduced atherosclerotic lesions after being immunized with malondialdehyde-modified LDL, in spite of elevated LDL cholesterol levels [83]. Another example of anti-atherogenic effect of autoantibodies against oxLDL is the marked reduction in neointimal area after balloon injury in cholesterol-fed rabbits despite severe hypercholesterolemia [84].

Path C: In the presence of excess generation of ROS and/or insufficient antioxidant capacity, or both, oxidative modification will proceed to the propagation phase (see Figure 1) resulting in the conversion of all polyunsaturated fatty acids to lipid hydroperoxides and subsequent irreversible modification of the lipoprotein particle, including fragmentation of the apolipoprotein moieties. The damaged lipoprotein particle would be taken up by the scavenger receptor, ultimately transforming monocyte-derived macrophages into foam cells.

**Figure 4:**
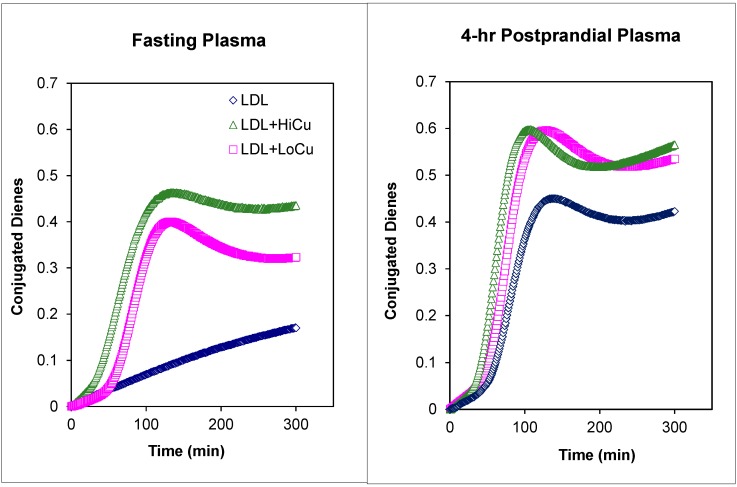
Meal-induced changes in oxidative susceptibility of LDL isolated from fasting versus LDL isolated from postprandial plasma of a patient with T2DM under dietary management. Blue symbols: LDL in the absence of Cu^2+^ as catalyst. Green symbols: LDL in the presence of high Cu^2+^ concentrations (9.0 mmol Cu^2+^ per 45 mg of LDL-C). Pink symbols: LDL in the presence of low Cu^2+^ concentrations (4.5 mmol Cu^2+^ per 45 mg of LDL-C).

Figure 4 illustrates the changes in oxidative susceptibility for LDL isolated from a patient with T2DM under control (normal fasting plasma lipids and glucose, HbA1c of 7.8) with insulin glargine (Lantus^®^; Sanofi-Aventis, Paris, France) administered subcutaneously once daily at bedtime. Postprandial plasma was obtained at 4-h following the consumption of a standardized commercially available test meal breakfast containing 39 g fat, 78 g carbohydrate, 24 g protein and 750 kcCal (1 bacon, egg, and cheese biscuit + 1 hash brown patty + 8 fl oz (237 mL) of orange juice) [85].

In the absence of Cu^2+^ (blue symbols), the formation of conjugated dienes in fasting LDL was slow for 300 min without the characteristic propagation phase (see Figure 1A). In contrast, even in the absence of Cu^2+^ to initiate the oxidation, LDL isolated from postprandial plasma underwent spontaneous oxidative modification with a lag time of 101.4 min. This is suggestive of greater presence of ROS on postprandial plasma as compared to fasting plasma. In the presence of low concentrations of Cu^2+^ (4.5mmol Cu^2+^ per 45 mg of LDL-C), the lag times for fasting and postprandial LDL were comparable, 21.1 *versus* 20.4 min, respectively. These lag times are short when compared to lag times obtained in our laboratory for normolipidemic controls without any CVD risk factors (range: 45–85 min). In the presence of high concentrations of Cu^2+^ (9.0 mmol Cu^2+^ per 45 mg of LDL-C), the lag times for both fasting and postprandial plasma were shorter that those obtained with low Cu^2+^ concentrations. The lag time for fasting LDL was slightly longer than that observed for postprandial plasma, 8.0 *versus* 5.4 min, respectively. Details for the *in vitro* oxidative susceptibility assay have been published previously [59].

**Figure 5:**
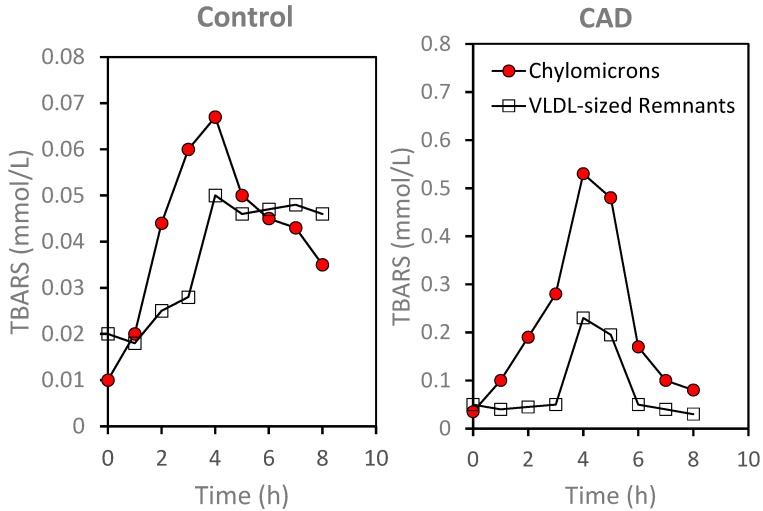
Time-dependent changes in lipid hydroperoxides as assessed by TBARS in lipoprotein fractions isolated by density ultracentrifugation following meal consumption. Note the differences in scale for the *y*-axis. Closed circles: Chylomicron fraction. Open squares: VLDL-sized remnants (for details on the isolation methods see [78]).

The increased presence of ROS on plasma lipoproteins following meal consumption was suggested by increases in lipid hydroperoxides, as estimated by thiobarbituric active substances (TBARS), on lipoprotein fractions (Figure 5). Chylomicrons and VLDL-sized remnants were isolated from plasma by sequential density ultracentrifugation as previously described [77] and TBARS were determined by fluorescence spectroscopy using a modified method of Yagi [86,87]. As illustrated in Figure 5, TBARS levels were lower in the control subject as compared to the patient with documented CAD for both chylomicrons, 0.01 *versus* 0.03 mmol/L, respectively, and for VLDL-sized remnants, 0.02 *versus* 0.05 mmol/L. Following meal consumption, there were increases in TBARS levels in both chylomicrons and VLDL-sized remnants for control as well as for the CAD patient. Relative to fasting level, there was an approximately 15-fold increase in TBARS level in the chylomicron fraction at 4 h after meal consumption in the CAD subject, as compared to only a five-fold increase in the control subject. When compared to fasting levels, TBARS levels in the VLDL-sized remnants at 4 h after the test meal was 4.6-fold higher for the CAD subject and 2.5-fold higher for the control subject. While the difference in meal-induced changes in TBARS on isolated plasma lipoproteins need to be confirmed, similar changes were observed in a small group of four CAD patients and three normolipidemic controls.

## 6. Oxidative Stress: Protective Response Leading to Disease

Among the recommendations that have been stressed as part of a healthy lifestyle are physical activity and dietary regimens enriched in polyunsaturated fatty acids. Both of these, however, are associated with significant generation of ROS. It has been suggested that although excess production of ROS may lead to oxidative stress causing damage to lipids, proteins and nucleic acids, at moderate levels ROS play an important role as regulatory mediators in signaling processes.

Generation of ROS and reactive nitrogen species (RNS) by skeletal muscle has been well documented during both the resting state and contractile activity when production is increased [88,89,90]. Many of the ROS-mediated responses actually protect the cells against oxidative stress and re-establish redox homeostasis [91]. For instance, exercise conditioning has been reported to induce ROS production which affects transcription factors such as nuclear factor kappa B (NF-κB), activator protein 1 (AP1) [92,93], and mitogen-activated protein kinases (MAPKs) [94] with the net result being a reduction in oxidative stress [95]. Without proper conditioning, physical activity that requires high-power output levels, such as sprint exercise, have been reported to cause oxidative stress leading to muscle damage [96].

With respect to dietary polyunsaturated fatty acids (PUFA), the data reviewed here would support an analogous process. Moderate consumption of polyunsaturated fatty acids with each meal would allow seeding of plasma TRL with ROS which could induce an immune response by facilitating the production of antibodies specific against oxidized epitopes on plasma lipoproteins. In other words, with each meal, we are immunizing ourselves against oxidized epitopes and thus the beneficial effects of polyunsaturated fatty acids [66,68,69,84]. A review of the literature on the use of oxidative epitopes to develop a vaccine against atherosclerosis is available [97]. In the present schema, we are proposing that autoimmunization would occur *in vivo* by providing intermittent flux of oxidizable PUFA as part of the diet [68,69]. Under normal circumstances, we would expect low concentrations of oxLDL to be bound to circulating antibodies and removed via the Kupffer cells and rapidly removed. Thus, a priori, we would expect high titers of autoantibodies against oxidatively modified LDL to be associated with reduced atherosclerotic risk. This is not demonstrated, however, in a number of case-control studies which have reported higher autoantibody titers in patients with documented CAD [98,99]. It is possible that, in patients with existing atherosclerotic disease, the presence of monocyte-derived macrophages in the arterial wall with up-regulated scavenger receptors would actually shift the balance to accumulation oxLDL and LDL-IgG complexes and subsequent transformation into foam cells. In this instance, consumption of highly oxidizable PUFA may not be beneficial unless accompanied by significant reductions in inflammatory/oxidative status.

## 7. Discussion

In summary, in spite of the well-documented efficacy of lipid-lowering statins and significant reductions in cardiovascular events, atherosclerotic progression remains a problem in some individuals [100,101]. For instance, in the landmark clinical trial CARE, Cholesterol And Recurrent Events with a significant 24% reduction in risk, the frequency of the primary endpoint was 10.2% in the treated group as compared to 13.2% in the placebo group [102].

There is an increasing interest in understanding other processes that might contribute to this progression, the so-called residual risks. It has been suggested that inflammation, as assessed by hsCRP and lipoprotein-associated phospholipase A_2_, may be contributory factors [103,104,105]. In a survey of 30 novel biomarkers, Blankenberg and colleagues have identified hsCRP as a key player [106]. The clinical significance of these markers of inflammation remains, however, unclear [107].

Plasma levels of oxidized lipoproteins, in particular oxLDL, have also been suggested to be an independent contributing factor [82,108]. A number of prospective studies have suggested a potential role of oxidized lipoproteins, though the exact measure of oxidation is not well understood [82,108]. While progression study in nonhuman primates does suggest increases in the plasma levels of oxidized phospholipids (oxPL), the contents of oxPL in the atherogenic apoB-containing lipoproteins was actually decreased by 31% [109]. In the same report, reduction of total cholesterol from 418 to 64 mg/dL with concomitant reduction of apoB from 115 to 18 mg/dL as part of the regression study resulted in a 47% increase in oxPL contents in apoB-containing lipoproteins [109]. In a small group of hypercholesterolemic patients, reductions in total cholesterol (19%–32%) with concomitant reduction in apoB (15%–31%) were accompanied by reductions in oxLDL (23%–35%) using one assay and by increases in oxPL contents of apoB-containing lipoproteins (20%–25%) using a different assay [110]. In the REVERSAL trial (Reversal of Atherosclerosis with Aggressive Lipid Lowering) in human subjects, in spite of 39% reductions in apoB with atorvastatin and lack of disease progression as assessed by percentage changes in atheroma volume using intravascular ultrasound [111], there was significant increase in oxPL contents in apoB-containing lipoproteins (48%), and malondialdehyde contents in apoB-containing lipoproteins (21%) [112]. Neither fasting levels of antibodies against MDA-modified LDL nor fasting levels of immune complexes were predictive of the observed changes in lipids and atheroma volume [112].

The present review presents data in support of a functional assay of oxidative susceptibility of plasma LDL both in the fasting state and in response to a physiological bout of oxidative stress as potential risk factor contributing to the progression of atherosclerotic disease. We have shown that LDL oxidative susceptibility is increased during postprandial lipemia in patients with metabolic syndrome [59], a finding consistent with an earlier report by Anderson and colleagues [113]. They also noted meal-induced increases in TBARS levels [113] which have been linked to coronary heart disease risk [114] as well as future cardiovascular events in patients with stable coronary artery disease [115]. Together with the transient increases in TBARS during postprandial lipemia (Figure 5), the increase in oxidative susceptibility of plasma lipoproteins during postprandial lipemia (Figure 4) may account for increased production of oxidatively modified lipoproteins. While we have not presented data demonstrating that this meal-induced increase in oxidative susceptibility of plasma LDL is transient, we have reported that the meal-induced generation of oxidatively modified epitopes is a transient process with fasting baseline levels being observed by 6–8 h after meal consumption [80,81]. A possible explanation is that LDL particles with higher TBARS contents are more susceptible to oxidative modification and would be more likely to become oxidatively modified and thus contribute to the disease process to a greater extent. Direct data linking changes in oxidative susceptibility of plasma lipoproteins and cardiovascular disease risk would be needed to support this concept.
